# SF3B1 as therapeutic target in *FLT3*/ITD positive acute myeloid leukemia

**DOI:** 10.1038/s41375-021-01273-7

**Published:** 2021-05-17

**Authors:** Inge van der Werf, Anna Wojtuszkiewicz, Huilan Yao, Rocco Sciarrillo, Manja Meggendorfer, Stephan Hutter, Wencke Walter, Jeroen Janssen, Wolfgang Kern, Claudia Haferlach, Torsten Haferlach, Gerrit Jansen, Gertjan J. L. Kaspers, Richard Groen, Gert Ossenkoppele, Jacqueline Cloos

**Affiliations:** 1 Dept. of Hematology, Amsterdam University Medical Center, VU University Medical Center, Cancer Center Amsterdam, Amsterdam, The Netherlands; 2 H3 Biomedicine, Inc, Boston, MA USA; 3 MLL Munich Leukemia Laboratory, Munich, Germany; 4 Amsterdam Rheumatology and Immunology Center, Amsterdam University Medical Center, VU University Medical Center, Amsterdam, The Netherlands; 5 Princess Máxima Center for Pediatric Oncology, Utrecht, The Netherlands; 6 Department of Pediatric Oncology, Emma Children’s Hospital Amsterdam, Amsterdam UMC, Vrije Universiteit Amsterdam, Amsterdam, The Netherlands

**Keywords:** Acute myeloid leukaemia, Targeted therapies

## To the Editor:

Recently, significant advances have been made in the development of splicing modulators for therapeutic purposes. In this respect, several studies demonstrated that acute myeloid leukemia (AML) cells carrying spliceosome mutations are preferentially sensitive to Splicing Factor 3B subunit 1 (SF3B1) modulation [1–3]. Whereas ~15% of AML patients have mutations in this class of genes, disruption of splicing appears to be a global phenomenon in hematological malignancies [4]. Therefore, we aim to identify additional patient subgroups which will benefit from these emerging modulators.

Towards this goal, we assessed the response to splicing modulation in a set of AML samples with different molecular aberrations. This included both cell lines as well as cryopreserved mononuclear cells isolated from diagnostic bone marrow samples. Cells were exposed to the SF3B1 modulator E7107, a stable analogue of Pladienolide B, and the newly developed H3B-8800. Subsequently, in vitro and ex vivo cytotoxicity measurements, functional assays and differential expression analyses were performed (supplementary materials and methods).

While growth of all cell lines was inhibited at nanomolar concentrations, cell lines carrying an internal tandem duplication (ITD) in the FMS-like tyrosine kinase 3 (*FLT3*) gene were particularly sensitive (Supplementary Fig. S1A). This effect in *FLT3/*ITD^pos^ cells was related to induction of cell cycle arrest (Supplementary Fig. S1B) with a concomitant shift in the splicing pattern of *MCL1* towards its pro-apoptotic variant (Supplementary Fig. S1C, D) upon relatively low drug concentrations compared to *FLT3/*ITD^neg^ cells. *FLT3/*ITD is a recurrent aberration in AML, which results in activation of downstream signaling pathways involved in proliferation, differentiation and apoptosis [5]. To date, patients with a high allelic ratio (AR; mutated over wild type) have been classified as high risk and are eligible for FLT3 inhibitors such as midostaurin. However, this drug only modestly improved survival in *FLT3/*ITD^pos^ AML patients [6], hence, there is still an unmet need for new drugs to treat this aggressive type of leukemia.

**Fig. 1:**
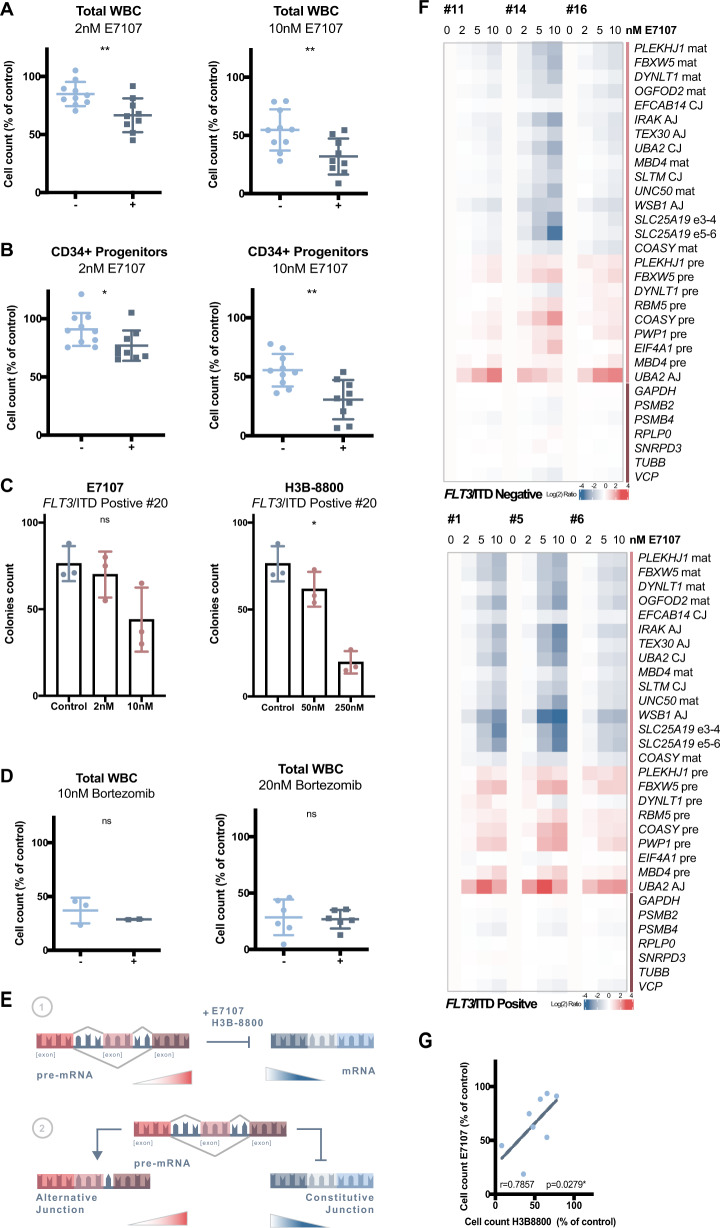
Response of primary AML cells with or without *FLT3*/ITD to splicing modulation. Ex vivo AML patients’ cells taken at diagnosis were selected based on their *FLT3* mutation status and the absence of any other aberrations based on our diagnostic panel. Light blue dots (−) represent *FLT3*/ITD^neg^ samples whereas dark blue dots (+) represent *FLT3*/ITD^pos^ specimens. **A**, **B** Cells were treated for 48 h with indicated doses of E7107. Cell count of the total white blood cell populations (WBC) was determined using Flow Cytometry. Progenitors were identified based on CD34 expression. Cell counts are depicted as percentage of untreated cells. **C** Clonogenic capacity of *FLT3*/ITD^pos^ cells derived from a primary bone marrow sample upon treatment with E7107 or H3B-8800. *P* values are based on Kruskas Wallis test. **D** Cells were treated for 48 h with indicated doses of Bortezomib. Cell count of the total white blood cell populations (WBC) was determined using Flow Cytometry. Cell counts are depicted as percentage of untreated cells. *P* values are based on Mann–Whitney U test. **E** Schematic of the effect of splicing modulation on both pre-mRNA as well as mature mRNA levels presented in (**G**). In short, splicing modulation via E7107 or H3B-8800 results in decreased production of mature mRNA transcripts (mat; blue) and concomitant accumulation of pre-mRNA transcripts (pre; red). In addition, alternative junctions (AJ; red) of certain genes are preferred upon splicing modulation resulting in decreased expression of constitutive spliced mRNAs (CJ; blue). (**F**) Representative heatmap of 3 patients samples depicting dose-dependent modulation for both mature as well as pre-mature mRNA markers in *FLT3*/ITD^neg^ (left) and *FLT3*/ITD^pos^ (right) cells. (**G**) Correlation between the response of AML patients’ cells to 10 nM E7107 or 250 nM H3B-8800. A strong association between both splicing modulators as indicated by high Spearman’s rho correlation coefficient (R).

Our hypothesis of preferential sensitivity to SF3B1 modulation in *FLT3/*ITD^pos^ samples was further tested in an ex vivo setting. For this purpose, cells taken at diagnosis were selected based on their *FLT3* mutation status and the absence of other aberrations based on our diagnostic panel (Supplementary Table S1). Corroborating in vitro results, the total population of white blood cells (WBC), and in particular the CD34 + progenitor population, within the leukemic bone marrow was more affected in *FLT3/*ITD^pos^ samples compared to *FLT3/*ITD^neg^ samples upon incubation with either E7107 or H3B-8800 (Fig. 1A and B, Supplementary Fig. S2A, B). Beside cell count, clonogenicity was found to be affected upon splicing modulation in *FLT3*/ITD^pos^ cells as well (Fig. 1C). To verify the selectivity of our findings, cells were simultaneously treated with the proteasome inhibitor Bortezomib, a compound whose mechanistic effects are not splicing dependent. Accordingly, no differences in response between *FLT3*/ITD^pos^ and *FLT3*/ITD^neg^ cells were observed with this compound (Fig. 1D).

Next, the response to splicing modulation was reflected in the dose-dependent splicing perturbation of a selected panel of mRNA transcripts (Fig. 1E, F, Supplementary Fig. S2C; Nanostring). Overall, decreased production of mature mRNA transcripts and concomitant accumulation of pre-mRNA transcripts seems to be more pronounced in *FLT3/*ITD^pos^ samples suggesting increased target engagement [7]. In addition, splicing of mature mRNA as well as pre-mRNA transcripts correlated with ex vivo response confirming enhanced splicing perturbation in *FLT3/*ITD^pos^ cells (Supplementary Fig. S2D, E). Importantly, this effect seems to be irrespective of other molecular aberrations (Table S1) due to their absence in *FLT3*/ITD^pos^ samples. Furthermore, response rates to both splicing modulators were highly correlated (Fig. 1G) confirming the general high activity of splicing modulators in *FLT3/*ITD^pos^ cells.

**Fig. 2:**
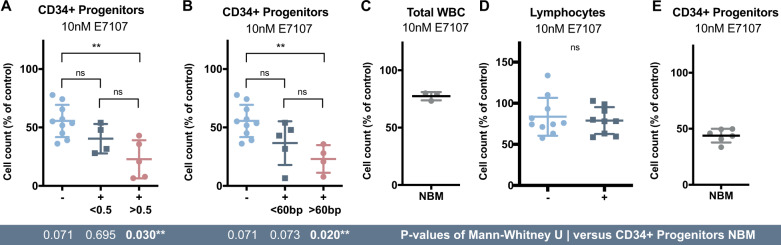
Preferential sensitivity to splicing modulation of *FLT3*/ITD^pos^ AML patients with high AR or long ITD length. **A** Cell counts of CD34 positive cells within bone marrows of patient samples grouped based on their *FLT3*/ITD allelic ratio according to the ELN 2017 following treatment with 10 nM E7107. **B** Cell counts of CD34 positive cells within bone marrows of patient samples grouped on their ITD size. Cut off was based on mean size of ITD length of selected patients. **C** Cell count of the total white blood cell population within healthy bone marrow was assessed upon treatment. **D** Cell counts of lymphocytes within AML bone marrow samples were identified based on CD45, CD34, and CD7 expression. **E** Cell counts of CD34 positive cells within healthy bone marrow samples was determined upon treatment with 10 nM E7107 for 48 h. Cell counts were plotted as a percentage of untreated cells. The Mann–Whitney U test was used to compare response levels between different groups of patients or normal bone marrow.

In order to further explore the role of the *FLT3/*ITD in the sensitivity to E7107, we evaluated samples with a low (<0.5) or high (>0.5) AR based on the ELN2017 classification [8, 9]. CD34 + cells within the leukemic bone marrow of *FLT3/*ITD^pos^ samples with a high AR responded significantly better to splicing modulation as compared to *FLT3/*ITD^neg^ specimens (*p* = 0.03; Fig. 2A, Supplementary Fig. S3A, Supplementary Table 1). In addition, we found a gradual increase of response with increased AR which suggests a direct link of response to this aberration. The size of the ITD was also demonstrated to have prognostic significance in AML patients [10]. Concordantly, our data showed that the size of the ITD was associated with response (p = 0.02; Fig. 2B, Supplementary Fig. S3B, Supplementary Table 1). Thus, both the AR and ITD length seem to be important determinants of the efficacy of splicing modulation within this specific subgroup of AML patients.

To determine the therapeutic index, we examined total WBC counts within healthy bone marrow upon splicing modulation. These were not affected upon treatment with either E7107 or H3B-8800 (Fig. 2C; Supplementary Fig. S3C). Accordingly, lymphocytes count within all AML patient samples were not impacted by splicing modulation (Fig. 2D; Supplementary Fig. S3D). However, the subpopulation of CD34+ cells derived from healthy bone marrow was negatively affected by both splicing modulators (Fig. 2E; Supplementary Fig. S3E). This finding highlights the importance of selecting particularly sensitive cells, such as *FLT3*/ITD positive leukemia with high AR and/or long ITD length, together with assessing the right dose to avoid toxicity to non-malignant cells.

Having established high sensitivity in *FLT3/*ITD^pos^ patients for SF3B1 modulation, we next hypothesized that the downstream effectors of this aberration could explain the identified differences in sensitivity. Previously, oncogenic MYC activation was shown to confer stress on splicing machinery via upregulation of *PRMT5*, which makes the spliceosome an attractive target in MYC-driven cancers [11]. In *FLT3/*ITD^pos^ cells enrichment of c-MYC gene sets has been reported [12]. In addition, cells with either high *MCL1* or *BCL2A1* expression were shown to be preferentially sensitive to E7107 and elevated *MCL1* levels were demonstrated in *FLT3/*ITD^pos^ cells [13, 14]. To assess expression of these genes in *FLT3/*ITD^pos^ and *FLT3/*ITD^neg^ samples we used both qPCR as well as RNAseq data. Increased expression of none of the gene candidates was confirmed in *FLT3/*ITD^pos^ samples in our dataset (Supplementary Fig. S4, S5A, B). In fact, we found significantly decreased expression of *MYC* in *FLT3/*ITD^pos^ patients. In addition, we did not identify differential expression of MYC target genes which indicates there are no differences in post-transcriptional regulation either (data not shown). Functionally, we did identify differences in apoptosis upon treatment. We found that *FLT3/*ITD^pos^ cells presented increased markers of apoptosis upon treatment as well as subtle increase of pro-apoptotic MCL1-S, which was correlated with response (Supplementary Fig. S5C-E). Yet, this finding seems to be particularly pronounced when using relatively high concentrations of E7107. Therefore, apoptosis induction does not seem to reflect the cause, but rather the consequence of hypersensitivity of *FLT3/*ITD^pos^ cells to splicing modulation.

Notably, we did find significantly increased expression of *SF3B1* in patients with high AR or long ITD length in our dataset (Supplementary Fig. S6A), although, *FLT3/*ITD^pos^ patients could not be clustered based on expression of genes involved in splicing regulation (Supplementary Fig. S6B). In addition, response rates to SF3B1 inhibition were not correlated with *SF3B1* expression levels (Supplementary Fig. S6C).

Interestingly, we did observe a drastic decrease of FLT3 RNA and protein levels upon 24 h of incubation with E7107 (Supplementary Fig. S7). However, downregulation does not seem to be derived from aberrant splicing since we did not identify changes in mRNA length suggesting lack of altered splicing (data not shown). Splicing modulation is known to occur rapidly following treatment [3] while FLT3 downregulation was only detected after 24 h, which suggest that the effect on FLT3 expression to be indirect. These findings, together with the that observation that cells with long ITD length and high AR are particularly sensitive to splicing modulation, suggests that hypersensitivity of *FLT3/*ITD^pos^ samples to splicing modulation could rely on FLT3 gene expression regulation and subsequent downstream signaling events. This hypothesis is supported by the finding that long lTD length was associated with higher FLT3 kinase activity [10]. While kinase activity was not assessed in this study, as downregulation of FLT3 is bound to abrogate FLT3 kinase activity, *FLT3/*ITD positivity was previously linked with increased ex vivo response to FLT3 inhibition by gilteritinib supporting the idea of dependency of these cells on FLT3 signaling [9].

Altogether, this study provides several lines of evidence that splicing modulation holds potential as novel therapeutic option for AML patients carrying *FLT3/*ITD with high AR and/or long ITD length, who currently suffer from poor treatment outcome. Future studies should further validate our findings in isogenic models and uncover the precise role of splicing in *FLT3/*ITD^pos^ patients. Especially SF3B1 expression levels in the context of high AR or long ITD length seem to be of interest.

## Supplementary information

Supplemental Files

Table S1
