# Author Correction: SF3B1 as therapeutic target in FLT3/ITD positive acute myeloid leukemia

**DOI:** 10.1038/s41375-021-01340-z

**Published:** 2021-07-08

**Authors:** Inge van der Werf, Anna Wojtuszkiewicz, Huilan Yao, Rocco Sciarrillo, Manja Meggendorfer, Stephan Hutter, Wencke Walter, Jeroen Janssen, Wolfgang Kern, Claudia Haferlach, Torsten Haferlach, Gerrit Jansen, Gertjan J. L. Kaspers, Richard Groen, Gert Ossenkoppele, Jacqueline Cloos

**Affiliations:** 1 Department of Hematology, Amsterdam University Medical Center, VU University Medical Center, Cancer Center Amsterdam, Amsterdam, The Netherlands; 2 H3 Biomedicine, Inc, Boston, MA USA; 3 MLL Munich Leukemia Laboratory, Munich, Germany; 4 Amsterdam Rheumatology and Immunology Center, Amsterdam University Medical Center, VU University Medical Center, Amsterdam, The Netherlands; 5 Princess Máxima Center for Pediatric Oncology, Utrecht, The Netherlands; 6 Department of Pediatric Oncology, Emma Children’s Hospital Amsterdam, Amsterdam UMC, Vrije Universiteit Amsterdam, Amsterdam, The Netherlands

**Keywords:** Acute myeloid leukaemia, Targeted therapies

Correction to: *Leukemia*

10.1038/s41375-021-01273-7

Despite carefully proofreading our submission, we missed a mistake in the affiliation of Wolfgang Kern, Claudia Haferlach and Torsten Haferlach. Instead of (2) H3 Biomedicine, their affiliation should be (3) MLL Munich Leukemia Laboratory, Munich, Germany.

