# Supplementary material for: SF3B1 as therapeutic target in FLT3/ITD positive acute myeloid leukemia
Source: Leukemia. 2021 May 17;35(9):2698–702. doi: 10.1038/s41375-021-01273-7 (PMC8410582; doi:10.1038/s41375-021-01273-7)
Supplement: Supplementary file 1 — Supplemental Files [file 41375_2021_1273_MOESM1_ESM.pdf]

SUPPLEMENTAL FILES

Material and Methods

Leukemic Cells

The human acute myeloid leukemia cell lines Molm13, Kasumi-1 and MM6 were obtained from DSMZ (Braunschweig, Germany) while MV4-11, KG1 and THP1 were obtained from ATCC (Manassas, Virginia, USA). All cell lines were maintained in RPMI-1640 medium (Gibco, Carlsbad, CA, USA) with either 10% or 20% fetal calf serum (FCS; Greiner Bio-One, Frickenhausen, Germany), 100 units/mL penicillin G (Gibco) and 100 ug/mL streptomycin sulphate (Gibco). Cryopreserved mononuclear cells isolated from diagnostic bone marrow samples were obtained from patients in clinical trials of the Dutch-Belgian Cooperation Trial Group for Hematology-Oncology (HOVON) in accordance with the declaration of Helsinki. Mononuclear cells were thawed in RPMI-medium supplemented with 20% FCS and incubated for 30 minutes with 100 µg/ml DNAse (Roche, Basel, Switzerland) and 10 mM $MgCl_2$. Subsequently, cells were washed and resuspended in RPMI-medium supplemented with 10% FCS, H-SCF (100ng/mL; Peprotech, Rocky Hill, NJ, USA), FLT3-L (100ng/mL; Peprotech) and IL3 (20ng/mL; Peprotech). Both cell lines as well as mononuclear fractions were cultured at 37°C incubator in a 5% CO_2 humidified atmosphere. Cells were repeatedly authenticated using STR profiling and tested for mycoplasma contamination.

Exposure to Spliceosome Modulators

Both E7107 and H3B-8800 were provided by H3 Biomedicine (Boston, MA, USA). Growth inhibitory effect of splicing modulators in AML cells lines was determined using CellTiter-Blue® Cell Viability Assay (Promega, Madison, WI, USA) upon drug incubations for 96 hours. In addition, exponentially growing cells were exposed to E7107 and H3B-8800 for 24 hours followed by flow cytometry-based cell cycle or apoptosis assessment and for 6 hours followed by RNA extraction (see below).

FLT3 Mutation Analysis

The status of *FLT3*/ITD and other gene mutations was determined according to the method described by Stone et al.[6] Data for patient samples treated with splicing modulation can be found in Supplemental Table S1.

RNA isolation and PCR analysis

Total RNA was extracted from leukemic cells using the RNA easy minikit (Qiagen, Venlo, The Netherlands). Reverse transcription was performed using 1ug of the obtained RNA and Moloney Murine Leukemia Virus reverse transcriptase (M-MLV; Invitrogen, Carlsbad, CA, USA) in a reaction buffer containing random hexamers (Roche) DNTPs (Roche) and ribonuclease inhibitor RNasin (Promega). Subsequently, splicing patterns of MCL1 were examined using PCR with 2x Reddy Mix PCR Master (Thermo Scientific, Waltham, MA, USA) following the manufactures instructions. The PCR products

were resolved on 2% agarose gels with Ethidium Bromide. Also, real-time PCR was used to asses splicing patterns of *MCL1-S*, *MCL1-L* and expression levels of *C-MYC*, *N-MYC* and *SF3B1*. The Lightcycler 480II Sybr Green 1 Master mix (Roche) were used for analysis according to manufacturer's protocol. Primer sequences are listed in Table S2.

Flow Cytometry

Samples were treated as described above. For cell cycle analysis, cells were permeabilized in 70% ethanol followed by 30 minutes incubation with RNAse (100ug/mL; Qiagen) and staining with Propidium Iodide (Thermo Fisher Scientific). Subsequently, cell count and Propidum Iodide (PI) staining were plotted to assess DNA content and distinguish G1, S, G2 and M phases. Apoptosis induction was analyzed using the Apoptest™-FITC kit (VPC Diagnostics, Hoeven, The Netherlands) and 7-AAD (BD Via-Probe™, BD Bioscience, San Jose, CA, USA). Furthermore, flow cytometry (BD FACSCelesta™) was used for cell count assessment and phenotypic analysis of mononuclear cell fractions. First, the quality of cells was assessed upon 24 hours drug incubation using the Apoptest™-FITC kit and 7-AAD. Samples with less than 50% viability were rejected at this time point. Upon 48 hours of incubation, cells were stained with 7-AAD. Absolute cell counts were determined based on the percentage of 7-AAD negative cells in a total volume of 70uL. Within the CD45dim cell populations progenitor cells were identified as CD34+ cells. The analysis of lymphocytes was performed upon staining with CD45-KO, CD34-BV421 and CD7-PE (BD Biosciences) and characterized by high CD45, high CD7 and low side scatter.
Expression levels of FLT3 (CD135) were determined using CD135-PE (BD Bioscience) antibodies. Cells were incubated with CD-135 PE antibodies, followed by the measurement of mean fluorescence intensity (MFI) of 7-AAD negative cells.

CFU Assay

Samples were thawed as described above. Subsequently, cells were washed and resuspended in RPMI plus 20% FCS (Gibco) and let to rest overnight at 37 °C and 5% CO_2. Subsequently, drug dilutions were prepared in Iscove's Modified Dulbecco's Media (IMDM; Gibco). Next, cells were washed and resuspended in IMDM containing E7107 or H3B-8800. Then cells were plated in methylcellulose (1 mL/well; Cat. H4435; STEMCELL Technologies Inc., Vancouver, BC, Canada) in concentrations varying from 10,000 to 50,000 cells/well, depending on colony forming capacity that was assessed in previous experiments. For each condition cells were plated in triplicate. The number of colonies was scored after two weeks of culture.

Nanostring Panel

Custom Nanostring panel was designed by H3 Biomedicine through Nanostring and validated at H3 Biomedicine based on splicing modulation using H3B-8800. nCounter Elements TagSet (84) and other

consumables were ordered from Nanostring (Seattle, WA). Customized oligonucleotide probe pools were ordered through Integrated DNA Technologies (Coralville, IA). Experimental procedure was done according to nCounter Elements XT Reagents User Manual and nCounter Analysis System User Manual for MAX. Briefly, hybridization master mix was generated using different oligonucleotide probe pools and TagSet84. For each reaction, 350ng of RNA was mixed with this mix, and incubated in a thermal cycler (67°C) for 18hrs followed by incubation at 4°C. Next, samples were run and analyzed. Raw counts were normalized using negative probes plus two times the standard deviation. In addition, positive control normalization was done using geometric mean of all the positive controls, with minimum threshold of 0.3 and maximum threshold of 3. In the end, content normalization was done using geometric mean of housekeeping genes, with minimum threshold of 0.1 and maximum threshold of 10. Subsequently, normalized gene counts were used for further data analysis.

RNA-Sequencing Analysis

The TruSeq Total Stranded RNA kit was used, starting with 250ng of total RNA, to generate RNA libraries following the manufacturer's recommendations (Illumina, San Diego, CA, USA). 2x100bp paired-end reads were sequenced on the NovaSeq 6000 with a median of 50 mio. reads per sample (Illumina, San Diego, CA, USA). Using BaseSpace's RNA-seq Alignment app (v2.0.1) with default parameters, reads were mapped with the STAR aligner (v2.5.0a,) to the human reference genome hg19 (RefSeq annotation). For gene expression analysis estimated gene counts were normalized applying Trimmed mean of M-values (TMM) normalization method of the edgeR package (PMID: 19910308). The resulting log2 counts per million (CPMs) were used as a proxy of gene expression. Genes with a CPM < 1 were filtered out.

Statistics

All statistical analyses were performed using Prism 8 Software as well as R version 3.6.3/R studio version 1.2.5, including ggplot2 (version 3.2.1) and ComplexHeatmap (version 2.2.0) packages.[1,2] The Mann-Whitney U test was used to compare cell counts between different subgroups of treated AML patient samples, differences in apoptosis induction, as well as mRNA expression levels. Associations between response rates, and response rates to expression levels were analyzed using Spearman's Rho test. In all analyses p-values at below 0.05 were considered statistically significant.

REFERENCES

1. Gu, Z., Eils, R. & Schlesner, M. Complex heatmaps reveal patterns and correlations in multidimensional genomic data. *Bioinformatics* **32**, 2847-2849, doi:10.1093/bioinformatics/btw313 (2016).
2. Valero-Mora, P. M. ggplot2: Elegant Graphics for Data Analysis. *2010* **35**, 3, doi:10.18637/jss.v035.b01 (2010).

SUPPLEMENTAL TABLES

Supplemental Table S1

See **SupplementalTable_S1.xlxs**

Supplemental Table S2

Primer	Sequence
**RT-PCR**	
MCL1 Forward	5'-GCCAAGGACACAAAGCCAAT-3'
MCL1 Reverse	5'-GCTCCTACTCCAGCAACACC-3'
**qPCR**	
MCL1-L Forward	5'-CTGCATCGAACCATTAGCA-3'
MCL1-L Reverse	5'-AAAAGCCAGCAGCACATTCC-3'
MCl1-S Forward	5'-GGCCTTCCAAGGATGGGTTT-3'
MCL1-S Reverse	5'-TATGCCAAACCAGCTCCTACTC-3'
SF3B1 Forward	5'-AAGAAGCCAGGATATCATGCC-3'
SF3B1 Reverse	5'-TTGTATTCATCTTCCCGGTC-3'
MYC Forward	5'-GGCTCCTGGCAAAAGGTCA-3'
MYC Reverse	5'-CTGCGTAGTTGTGCTGATGT-3'
N-MYC Forward	5'-TGATCCTCAAACGATGCCTTC-3'
N-MYC Reverse	5'-GGACGCCTCGCTCTTTATCT-3'

SUPPLEMENTARY FIGURE LEGENDS

Supplemental Figure S1. Sensitivity of AML cell lines to splicing modulation. Both MV4-11 and Molm13 represent *FLT3/*ITD^{pos} cell lines - MV4-11 (homozygous for *FLT3/*ITD) and Molm13 (heterozygous for *FLT3/*ITD). A.- The response of cell lines incubated with a range of E7107 (left) or H3B-8800 (right) concentrations for 96h (Top) and IC50 calculations based on dose response curves (Bottom; N=3). B.- Cells were incubated with 2nM E7107 (left) or 50nM H3B-8800 (right). Cell cycle phases are visualized in a histogram plot of the PI stain upon flow cytometric analysis, in addition the fraction of cells (%) in the S phase of the cell cycle with or without treatment is indicated (N=3). C.- Schematic representation of *MCL-1* splicing. D.- *MCL1* expression after incubation with a range of H3B-8800 concentrations. Pro (MCL-S) and anti-apoptotic (MCL1-L) splice variants are indicated.

Supplemental Figure S2. **Response of primary AML cells with or without *FLT3/*ITD to splicing modulation.** A, B - Cells were treated with different doses H3B-8800 for 48h. Cell count of the total white blood cell populations (WBC) was determined using Flow Cytometry. Progenitors were identified based on CD34 expression. Cell counts are plotted as a percentage of untreated cells. P-values are based on Mann-Whitney U test. C.- Splicing modulation via E7107 or H3B-8800 results in decreased production of mature mRNA transcripts (mat; blue) and concomitant accumulation of pre-mRNA transcripts (pre; red). In addition, alternative junctions (AJ; red) of certain genes are preferred upon splicing modulation resulting in decreased expression of constitutive spliced mRNAs (CJ; blue). Heatmap that presents dose-dependent modulation for both mature as well as pre-mature mRNA markers in *FLT3/*ITD^{neg} (left) and *FLT3/*ITD^{pos} (right) cells upon 4h treatment with H3B-8800. D, E. - Correlation of response rate upon treatment with either H3B-8800 (D) or E7107 (E) to expression of mRNA transcripts normalized to control. Light blue dots (-) represent *FLT3/*ITD^{neg} patients, dark blue dots represent *FLT3/*ITD^{pos} patients (+).

Supplemental Figure S3. Preferential sensitivity of *FLT3/*ITD^{pos} AML patients with high AR or long ITD length. A.- Patient samples were grouped on their *FLT3/*ITD allelic ratio according to the ELN 2017. B.- Patient samples were grouped on their ITD size. Cut off was based on mean size of ITD length of selected patients. C.- Cell count of the total white blood cell population within healthy bone marrow was assessed upon treatment D.- Lymphocytes within AML bone marrow samples were identified based on CD45, CD34 and CD7 expression. E.- Cell count of CD34 positive cells within healthy bone marrow samples was determined upon treatment. Cells were treated with 250nM H3B-8800 for 48h. Cell counts are plotted as a percentage of untreated cells.

Supplemental Figure S4. *MYC* expression in *FLT3/*ITD^{pos} subgroups of AML patients. A.- mRNA expression of *MYC* determined by RNAseq (left) or qPCR (right). Patient samples were grouped on their allelic ratio according to ELN2017 or ITD size (left). *FLT3/*ITD negative and positive cells with differences in sensitivity were subjected to qPCR (right). B.- mRNA expression of *PRMT5* determined by RNAseq. C.- mRNA expression of *N-MYC* determined by RNAseq (left) or qPCR (right). Patient

samples were grouped on their allelic ratio according to ELN2017 or ITD size (left). *FLT3/*ITD negative and positive cells with differences in sensitivity were subjected to qPCR (right).

Supplemental Figure S5. Apoptosis induction of primary AML cells upon E7107 treatment. A.- *MCL1* mRNA expression levels determined by RNA seq. B.- *BCL2L1* mRNA expression levels determined by RNAseq. C.- *Ex vivo* AML patients' cells, collected at diagnosis, were selected based on their *FLT3* mutation status. Cells were treated with various dosages of E7107 for 48h. Subsequently, flow cytometry-based apoptosis assays were performed. No differences in levels of apoptosis were measured in untreated cells or cells treated with 2nM or 5nM E7107, apoptosis induction is significantly higher in *FLT3/*ITD^{pos} cells upon incubation with 10nM E7107 (p-values based on Mann-Whitney U test). D.- Cells were incubated with a range of E7107 concentrations for 6h, followed by RNA isolation, cDNA synthesis and qPCR performed on *MCL1*. The ratio of pro-apoptotic *MCL1-S* to anti-apoptotic *MCL1-L* is plotted. E.- Association between response rates to E7107 (cell counts as percentage of control determined by Flow Cytometry) and the ratio of *MCL1-S* to *MCL1-L* (analyzed using qPCR). P-value determined by Spearman's rho test. Light blue dots represent *FLT3/*ITD^{neg} patients, dark blue dots represent *FLT3/*ITD^{pos} cells.

Supplemental Figure S6. Expression of *SF3B1* is significantly increased in *FLT3/*ITD^{pos} AML patients with high AR or long ITD length. A.- mRNA expression of *SF3B1* determined by RNAseq. B.- Heatmap of genes involved in splicing in *FLT3/*ITD^{pos} patients with high allelic ratio (AR). C. Lack of correlation between response rates to E7107 (determined by Flow Cytometry) and *SF3B1* expression levels (measured by qPCR). P-value determined by Spearman's rho test. Light blue dots represent *FLT3/*ITD^{neg} specimen, dark blue dots represent *FLT3/*ITD^{pos} cells.

Supplemental Figure S7. Splicing modulation specifically decreases FLT3 expression levels decrease in *FLT3/*ITD^{pos} AML cell lines. Both MV4-11 and Molm13 represent *FLT3/*ITD^{pos} cell lines, Kasumi-1 cells are negative for this aberration. Cells were incubated with different dosages of E7107 for 24h. Subsequently, FLT3 (CD135) expression levels were measured using Flow Cytometry. Results are plotted as percentage of expression levels in control cells (N=3). In addition, FACS plots of cells stained for FLT3 (CD135) after 24h treatment with either E7107 or Bortezomib are presented.

SUPPLEMENTAL FIGURES

S1

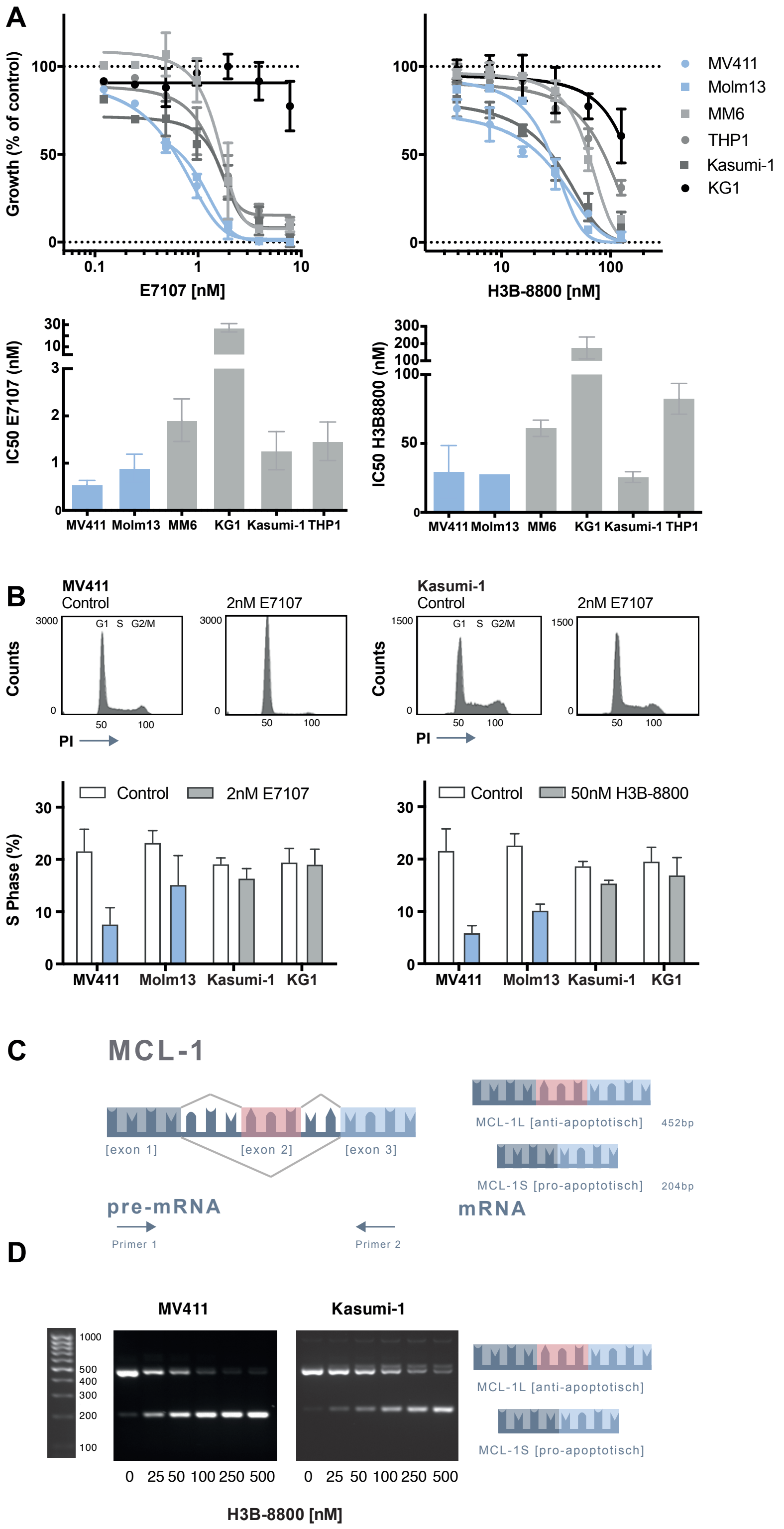
A
Growth (% of control)
E7107 [nM]
H3B-8800 [nM]
MV411
Molm13
MM6
THP1
Kasumi-1
KG1
IC50 E7107 (nM)
IC50 H3B8800 (nM)
MV411 Molm13 MM6 KG1 Kasumi-1 THP1
B
MV411
Control
2nM E7107
Kasumi-1
Control
2nM E7107
G1 S G2/M
Counts
PI
S Phase (%)
Control
2nM E7107
50nM H3B-8800
MV411 Molm13 Kasumi-1 KG1
C
MCL-1
[exon 1]
[exon 2]
[exon 3]
pre-mRNA
mRNA
MCL-1L [anti-apoptotisch] 452bp
MCL-1S [pro-apoptotisch] 204bp
Primer 1
Primer 2
D
MV411
Kasumi-1
1000
500
400
300
200
100
0 25 50 100 250 500
H3B-8800 [nM]
MCL-1L [anti-apoptotisch]
MCL-1S [pro-apoptotisch]

S2

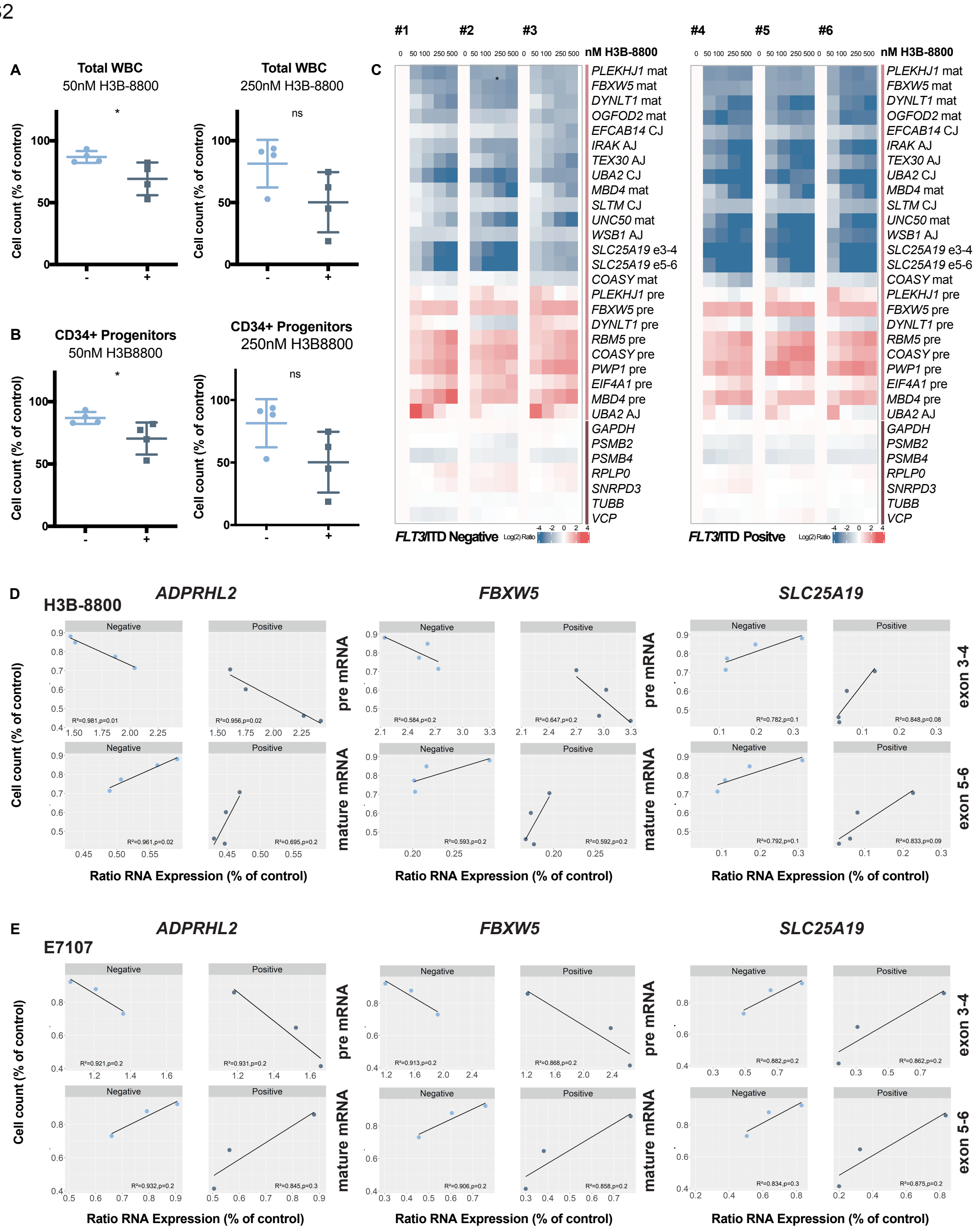
A
Total WBC
50nM H3B-8800
Total WBC
250nM H3B-8800
Cell count (% of control)
B
CD34+ Progenitors
50nM H3B8800
CD34+ Progenitors
250nM H3B8800
C
#1 #2 #3
nM H3B-8800
#4 #5 #6
PLEKHJ1 mat
FBXW5 mat
DYNLT1 mat
OGFOD2 mat
EFCAB14 CJ
IRAK AJ
TEX30 AJ
UBA2 CJ
MBD4 mat
SLTM CJ
UNC50 mat
WSB1 AJ
SLC25A19 e3-4
SLC25A19 e5-6
COASY mat
PLEKHJ1 pre
FBXW5 pre
DYNLT1 pre
RBM5 pre
COASY pre
PWP1 pre
EIF4A1 pre
MBD4 pre
UBA2 AJ
GAPDH
PSMB2
PSMB4
RPLP0
SNRPD3
TUBB
VCP
FLT3/ITD Negative
FLT3/ITD Positve
Log(2) Ratio
D
H3B-8800
ADPRHL2
FBXW5
SLC25A19
Negative
Positive
pre mRNA
mature mRNA
exon 3-4
exon 5-6
Ratio RNA Expression (% of control)
E
E7107
ADPRHL2
FBXW5
SLC25A19
Ratio RNA Expression (% of control)

S3

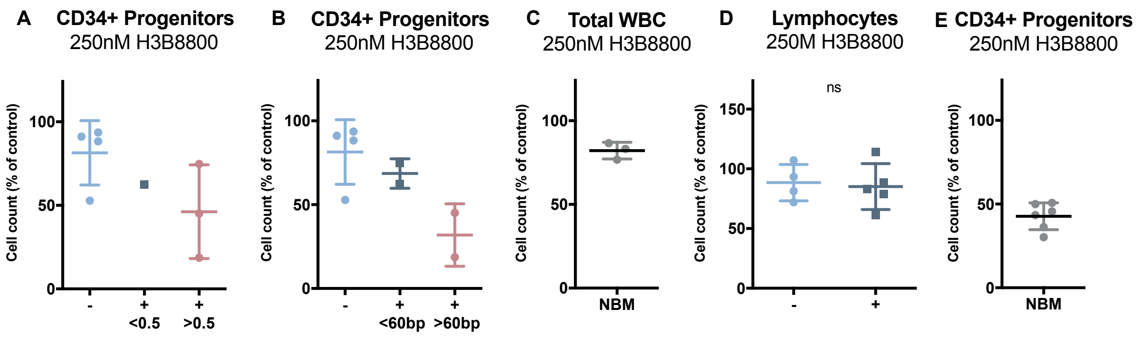
A
CD34+ Progenitors
250nM H3B8800
Cell count (% of control)
100
50
0
-
+
<0.5
+
>0.5
B
CD34+ Progenitors
250nM H3B8800
Cell count (% of control)
-
+
<60bp
+
>60bp
C
Total WBC
250nM H3B8800
NBM
D
Lymphocytes
250M H3B8800
ns
150
E
CD34+ Progenitors
250nM H3B8800
NBM

S4

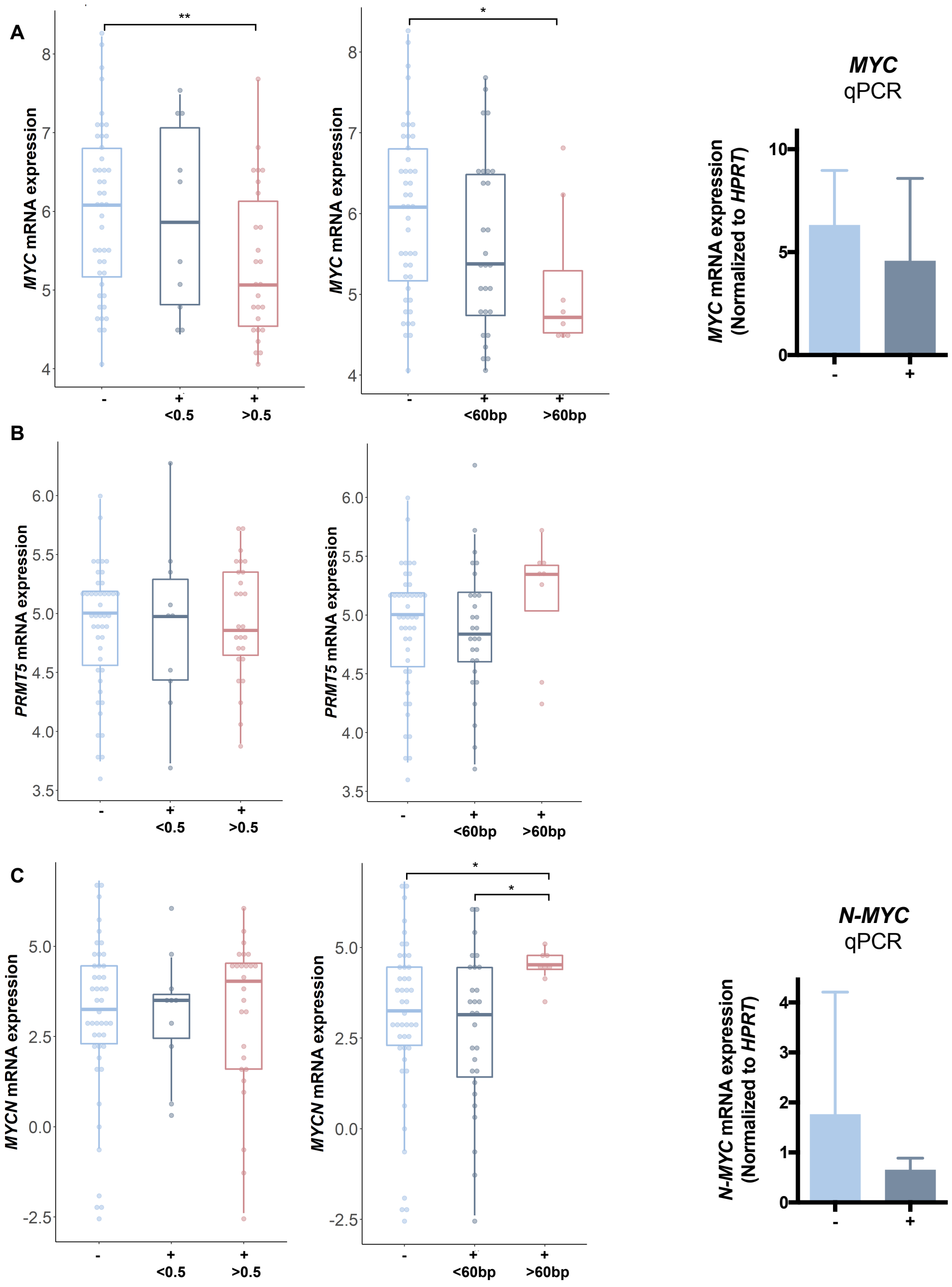
A
**
*
MYC mRNA expression
<0.5
>0.5
<60bp
>60bp
MYC
qPCR
MYC mRNA expression
(Normalized to HPRT)
B
PRMT5 mRNA expression
C
MYCN mRNA expression
N-MYC
qPCR
N-MYC mRNA expression
(Normalized to HPRT)

S5

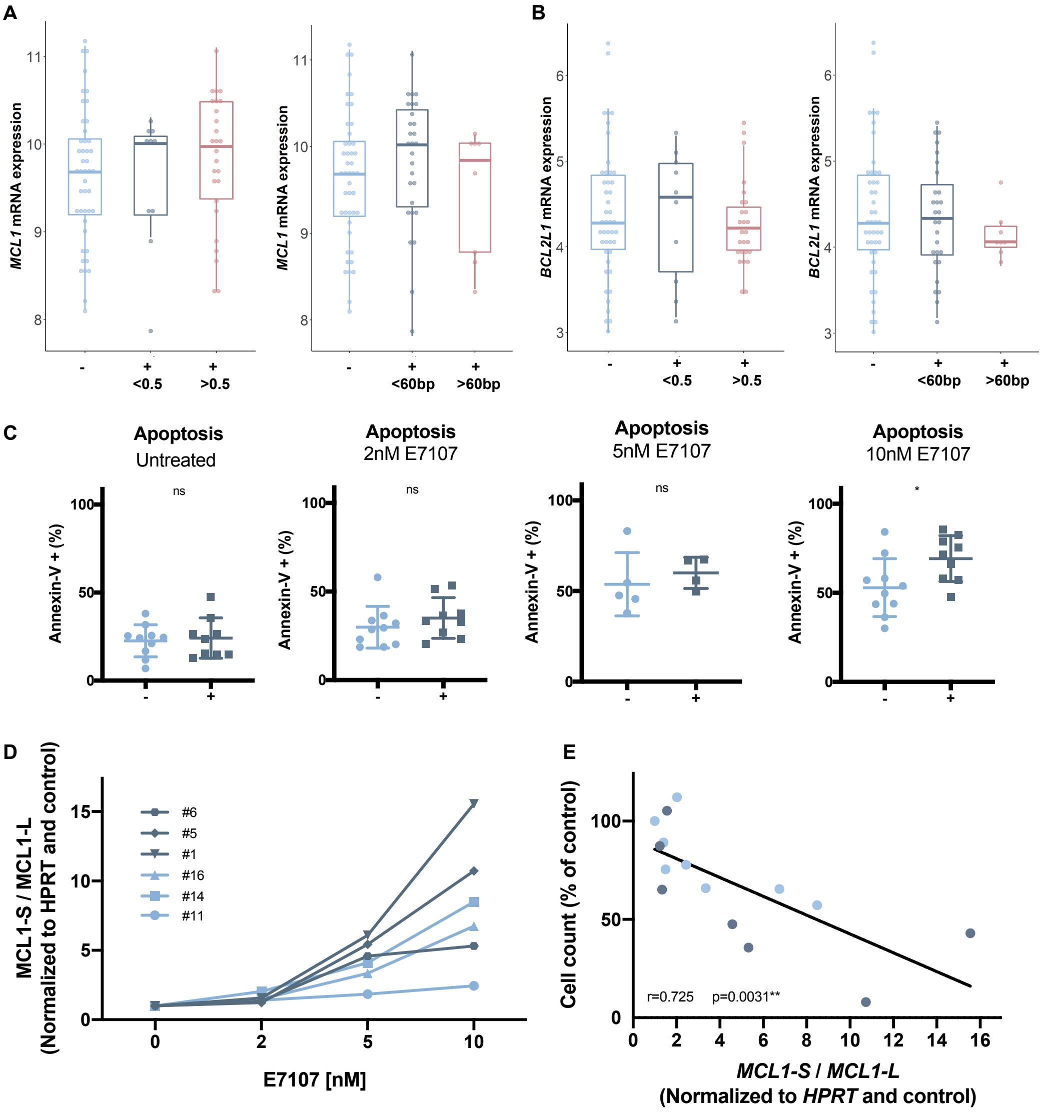
A
MCL1 mRNA expression
<0.5
>0.5
<60bp
>60bp
B
BCL2L1 mRNA expression
C
Apoptosis
Untreated
2nM E7107
5nM E7107
10nM E7107
Annexin-V + (%)
ns
*
D
MCL1-S / MCL1-L
(Normalized to HPRT and control)
#6
#5
#1
#16
#14
#11
E7107 [nM]
E
Cell count (% of control)
r=0.725
p=0.0031**
MCL1-S / MCL1-L
(Normalized to HPRT and control)

S6

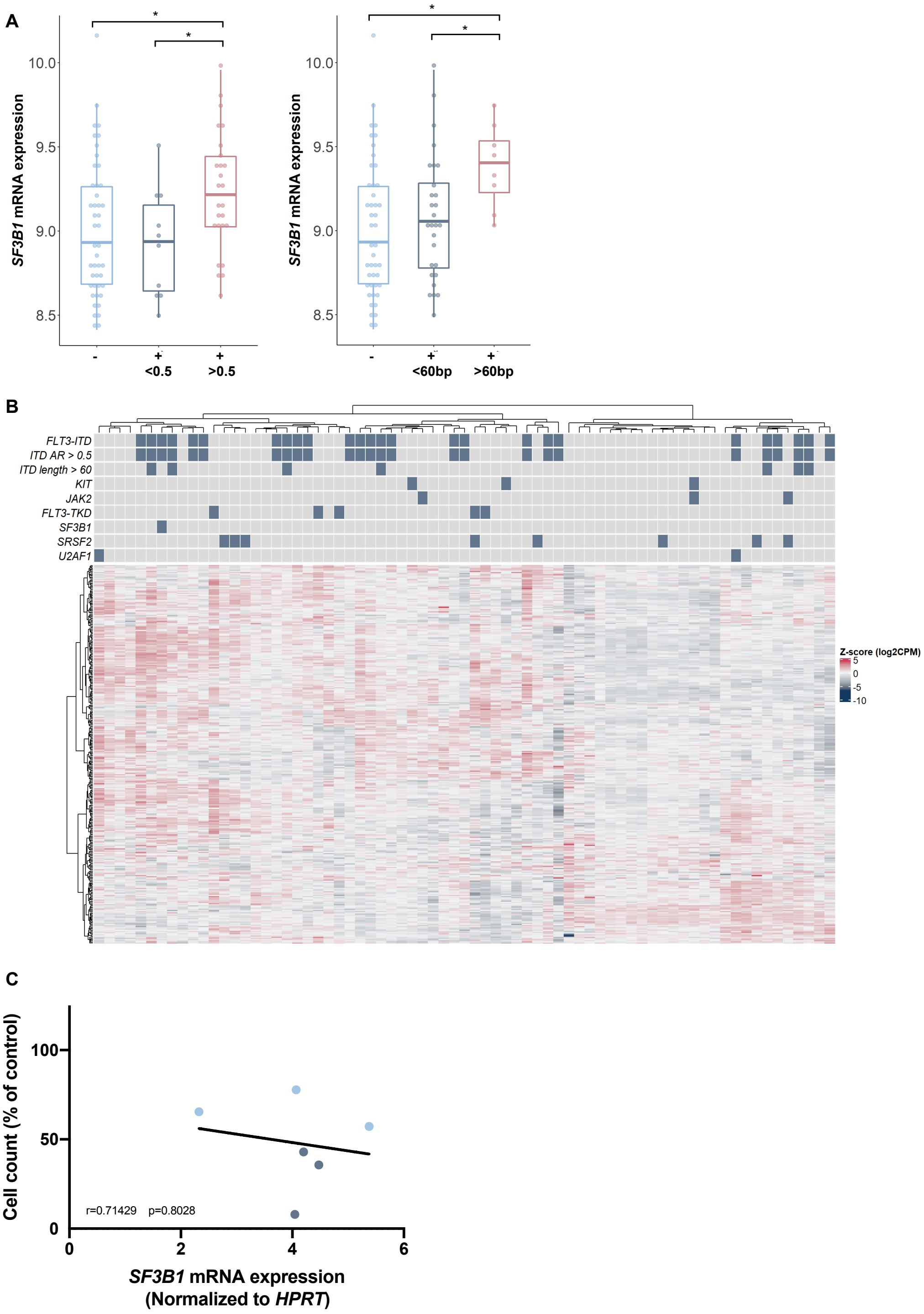
A
SF3B1 mRNA expression
10.0
9.5
9.0
8.5
-
+
<0.5
>0.5
<60bp
>60bp
*
B
FLT3-ITD
ITD AR > 0.5
ITD length > 60
KIT
JAK2
FLT3-TKD
SF3B1
SRSF2
U2AF1
Z-score (log2CPM)
5
0
-5
-10
C
Cell count (% of control)
100
50
0
r=0.71429 p=0.8028
SF3B1 mRNA expression
(Normalized to HPRT)
2
4
6

S7

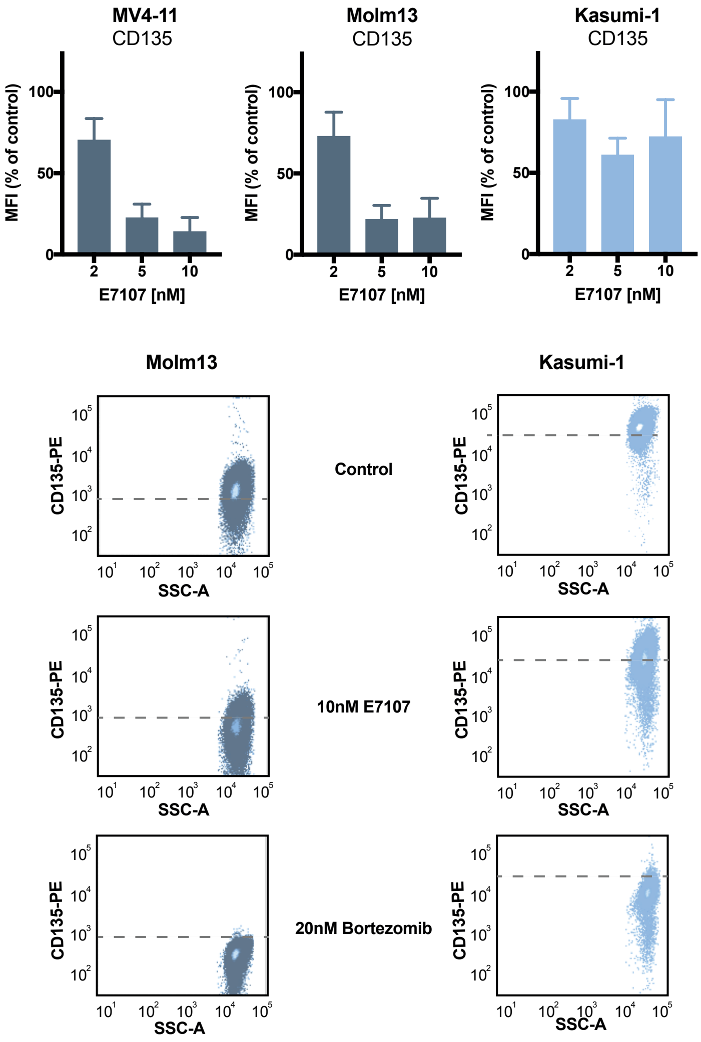
MV4-11
CD135
Molm13
CD135
Kasumi-1
CD135
MFI (% of control)
100
50
0
2
5
10
E7107 [nM]
Molm13
Kasumi-1
Control
10nM E7107
20nM Bortezomib
CD135-PE
SSC-A
